# Communication of gut microbiota and brain *via* immune and neuroendocrine signaling

**DOI:** 10.3389/fmicb.2023.1118529

**Published:** 2023-01-25

**Authors:** Kaja Kasarello, Agnieszka Cudnoch-Jedrzejewska, Katarzyna Czarzasta

**Affiliations:** Chair and Department of Experimental and Clinical Physiology, Laboratory of Centre for Preclinical Research, Medical University of Warsaw, Warsaw, Poland

**Keywords:** gut-brain axis, gut microbiota, HPA axis, immune system, vagus nerve

## Abstract

The gastrointestinal tract of the human is inhabited by about 5 × 10^13^ bacteria (of about 1,000 species) as well as archaea, fungi, and viruses. Gut microbiota is known to influence the host organism, but the host may also affect the functioning of the microbiota. This bidirectional cooperation occurs in three main inter-organ signaling: immune, neural, and endocrine. Immune communication relies mostly on the cytokines released by the immune cells into circulation. Also, pathogen-associated or damage-associated molecular patterns (PAMPs or DAMPs) may enter circulation and affect the functioning of the internal organs and gut microbiota. Neural communication relies mostly on the direct anatomical connections made by the vagus nerve, or indirect connections *via* the enteric nervous system. The third pathway, endocrine communication, is the broadest one and includes the hypothalamic-pituitary-adrenal axis. This review focuses on presenting the latest data on the role of the gut microbiota in inter-organ communication with particular emphasis on the role of neurotransmitters (catecholamines, serotonin, gamma-aminobutyric acid), intestinal peptides (cholecystokinin, peptide YY, and glucagon-like peptide 1), and bacterial metabolites (short-chain fatty acids).

## 1. Gastrointestinal tract as a living environment of the gut microbiota

The gastrointestinal tract (GI) is the external environment for our body. Therefore, the GI epithelium has to act as the protective barrier for the organism. Tight junctions connecting the epithelial cells and mucus produced by goblet cells are the physical barriers (Vancamelbeke and Vermeire, 2017). Moreover, enterocytes produce antimicrobial peptides (AMPs) which act directly on the bacterial cell membrane, causing its disruption and cell lysis (Chung and Raffatellu, 2018). Furthermore, the gut-associated lymphoid tissue (GALT) defends the organism against pathogens and, more importantly, maintains homeostasis in the GI tract (Mörbe et al., 2021). The molecules which allow our immune system to recognize potentially harmful microorganisms are the microorganism-associated molecular patterns (MAMPs), or in general for pathogens—the pathogen-associated molecular patterns (PAMPs). Such patterns are recognized by pattern recognition receptors (PRRs) present on the host cells of the innate immune system (Takeuchi and Akira, 2010; Wang and Wang, 2016). All these factors contribute to limiting the direct contact of the gastrointestinal epithelium with the microbiota. Therefore, the symbiotic gut microbiota which colonizes our intestines influences the GI epithelium, and interacts with the immune system, but in a limited scope (Hooper et al., 2012).

## 2. Gut microbiota–Characteristics

The term gut microbiota describes the microorganisms that exist in the GI, consisting mainly of bacteria, but also of archaea, fungi, and viruses (Quigley, 2017; Zhao and Elson, 2018; Zhao et al., 2021). The term microbiome, which is a broader concept that refers to microorganisms, their genomes, and the habitat they reside in, is often used instead of the term gut microbiota.

The colonization of the GI tract mostly takes place during birth, but there is evidence of trans-placental gut colonization (Quigley, 2017). It was shown that the gut microbiota profile in humans changes with the age of the host and is most stable in adulthood (Nicholson et al., 2012; Carabotti et al., 2015). In the healthy adult human, there are about 5 × 10^13^ bacteria (of about 1000 species) in the gut, mostly represented by the two phyla, *Firmicutes* and *Bacteroidetes* (Quigley, 2017; Zhao and Elson, 2018; Zhao et al., 2021), as well as less numerous *Actinobacteria*, *Proteobacteria*, *Fusobacteria*, *Verrucomicrobia*, and *Archaea* (Dubinski et al., 2021). The available data suggest that gender may have a significant influence on the composition and activity of gut microbiota (Makris et al., 2021). Clinical studies have shown that men when compared with women have an increased number of *Bacteroides* and *Prevotella*, as well as *Clostridia*, *Bacteroidetes*, and *Proteobacteria* phylum (Kovacs et al., 2011). However, the mechanisms explaining the above observation remain unclear (Bolnick et al., 2014).

The condition in which the balance between the commensal microflora and pathogenic microorganisms is maintained is called eubiosis (Iebba et al., 2016). Each deviation from the normal composition of gut microbiota (instability or a decrease in the number of *Firmicutes* and *Bacteroidetes*) and the accompanying violation of the intestinal epithelial barrier is referred to as dysbiosis, i.e., a deviation leading to an inflammatory state (Ahlawat et al., 2021; Dubinski et al., 2021).

The host organism creates favorable conditions for the development of gut microbiota, which in turn exert many positive effects on the host. Bacteria, through fermentation processes, produce metabolites such as lactates and short-chain fatty acids (SCFAs), which include acetate (C2), propionate (C3), and butyrate (C4) (Silva et al., 2020). These substances, synthesized in the gastrointestinal tract, can enter the circulation through the portal vein, and then *via* the blood to the peripheral tissues and organs, and also to the brain (Lerner et al., 2017). For example, butyrate is the most abundant of the SCFAs. It is synthesized in the large intestine by the following types of bacteria: *Clostridium*, *Eubacterium*, and *Butyrivibrio* (Bourassa et al., 2016; Czerwińska et al., 2021; Amiri et al., 2022; Table 1). It has been shown that butyrate has anti-inflammatory properties and has a positive effect on the intestinal epithelium, which may have a positive effect on the gut microbiota, increasing the number of the ENS cholinergic neurons, as well as modulating appetite through vagal and hypothalamic stimulation (Liu et al., 2018). Additionally, gut microbiota plays an important role in the synthesis of vitamins (K, riboflavin, biotin, nicotinic acid, pantothenic acid, pyridoxine, thiamine, and folic acid), and the metabolism of bile acids (Thursby and Juge, 2017). However, gut microbiota can also have adverse effects on the host organism, including the synthesis and release into the blood of lipopolysaccharide (LPS), also called endotoxin, which is a component of the cell wall of gram-negative bacteria and plays a key role in the initiation and progression of inflammation (Cani et al., 2012; Dubinski et al., 2021).

**TABLE 1:** The butyrate-producing bacteria.

Domain	Phylum	Class	Order	Family	Genus	Species
*Bacteria*	*Firmicutes*	*Clostridia*	*Eubacteriales*	*Clostridiaceae*	*Butyricicoccus*	*Butyricicoccus pullicaecorum*
					*Clostridium*	*Clostridium acetobutylicum* *Clostridium butyricum* *Clostridium saccharobutylicum* *Clostridium tyrobutyricum* *Clostridium orbiscidens* *Clostridium hathewayi* *Clostridium indolis* *Clostridium nexile*
					*Subdoligranulum*	*Subdoligranulum variabile*
				*Lachnospiraceae*	*Anaerostipes*	*Anaerostipes butyraticus* *Anaerostipes caccae* *Anaerostipes hadrus* *Anaerostipes rhamnosivorans*
					*Butyrivibrio*	*Butyrivibrio crossotus* *Butyrivibrio fibrisolvens* *Butyrivibrio proteoclasticus*
					*Coprococcus*	*Coprococcus catus* *Coprococcus comes* *Coprococcus eutactus*
					*Roseburia*	*Roseburia cecicola* *Roseburia faecis* *Roseburia hominis* *Roseburia intestinalis* *Roseburia inulinivorans*
					*Shuttleworthia*	*Shuttleworthia satelles*
				*Oscillospiraceae*	*Anaerotruncus*	*Anaerotruncus colihominis*
					*Faecalibacterium*	*Faecalibacterium prausnitzii*
					*Papillibacter*	*Papillibacter cinnamivorans*
					*Ruminococcus*	*Ruminococcus gnavus* *Ruminococcus obeum*
				*Eubacteriaceae*	*Eubacterium*	*Eubacterium cylindroides* *Eubacterium hallii* *Eubacterium limosum* *Eubacterium ramulus* *Eubacterium rectale* *Eubacterium ruminantium*
			*Thermoanaerobacterales*	*Thermosediminibacteraceae*	*Caldicellulosiruptor*	*Caldicellulosiruptor saccharolyticus*
		*Negativicutes*	*Veillonellales*	*Veillonellaceae*	*Megasphaera*	*Megasphaera elsdenii*

The gut microbiota has been shown to influence the host organism in three major pathways: (a) *via* immune systems, (b) *via* neurotransmitters, and (c) *via* microbial metabolites (Wang and Wang, 2016).

### 2.1. Gut microbiota and immune system interaction

As previously mentioned, the mucus layer reduces to a large extent the direct contact of the gut microbiota with the GI epithelium. Bacteria that reach deeper into the mucosal surface are sampled by the protrusions of the dendritic cells (DCs), which are the specialized antigen-presenting cells (APCs). Next, the DCs go to the mesenteric lymph node, where the sampled antigens are presented to T and B lymphocytes (Hooper et al., 2012).

In the state of eubiosis, physical barriers are intact and molecules such as IgA and AMPs, present in the gut lumen, provide the control mechanisms inhibiting the pathogen spreading. Moreover, symbiotic bacteria itself produces the AMPs against pathogenic strains (Iebba et al., 2016). Immune cells composing GALT present the tolerogenic phenotype as the result of the influence of transforming growth factor β (TGFβ) produced by the epithelial cells. TGFβ causes the differentiation of immune cells toward the anti-inflammatory tolerogenic phenotype. In addition, CD103 + DCs residing in the lamina propria produce anti-inflammatory interleukin 10 (IL-10), which further influences T and B cell differentiation (Siddiqui and Powrie, 2008; Moro-Sibilot et al., 2016; Pathak et al., 2020). B cells in the mesenteric lymph nodes produce the IgA antibodies against the bacterial antigens, and next go to the lamina propria and secrete the IgA into the intestinal lumen. Here, the IgA binds to the bacterial antigens and prevents bacterial translocation through the gut epithelium. In case of bacterial penetration under the epithelium, the resident macrophages and DCs in the lamina propria phagocyte the microorganisms (Hooper et al., 2012). In turn, naive T cells under the influence of the commensal bacteria antigens are differentiated into regulatory T cells (Tregs) (Lathrop et al., 2011). Such a mechanism underlies the mechanism of oral tolerance, which prevents the evoking of an immune response against dietary antigens and against symbiotic microflora. Furthermore, induced Tregs secrete the anti-inflammatory cytokines, IL-10, and TGF-β. When the pathogenic bacteria encounter GALT, naive T cells differentiate into Th1/Th17 subtypes, producing proinflammatory cytokines (Zhao and Elson, 2018). Moreover, CD103^+^ DCs also contribute to the production of retinoic acid (RA) from vitamin A, which is induced by the SCFAs obtained from dietary fibers. RA is also the factor inducing Tregs differentiation (Luu et al., 2017). In such a homeostatic state, tolerance to dietary antigens and antigens from commensal microflora is maintained.

On the other hand, during dysbiosis, pathogens and their harmful metabolites are recognized by the PRRs on the surface of the immune cells of the host, and an innate immune response is evoked. Enterotoxins released by pathogens, such as LPS, cause damage to the intestinal epithelium, which results in gut permeability and the entrance of pathogens into the circulation. The damage to the gut epithelium may also contribute to intestinal inflammation. Environmental factors, such as a western diet, antibiotics, stress, or injury may cause damage to the gut epithelium, allowing bacterial gut penetration, which induces an immune reaction (Cresci and Bawden, 2015; Lobionda et al., 2019). Mediators released by the immune cells during an inflammatory reaction enter the circulation to attract more immune cells. PAMPs released from damaged pathogens may also enter the circulation.

Two examples of PRRs are toll-like receptors (TLRs) and nucleotide-binding oligomerization domain (NOD)-like receptors (McCusker and Kelley, 2013). The interaction of PRRs and PAMPs results in the activation of intracellular signaling pathways, leading to nuclear factor kappa-light-chain-enhancer of activated B cells (NF-κB) translocation to the nucleus, and results in the activation of gene transcription for proinflammatory cytokines (e.g., interleukin-1, IL-1; tumor necrosis factor α, TNFα; interferon gamma, IFNγ) (McCusker and Kelley, 2013). Interestingly, the TLRs of the innate immune systems can distinguish between the MAMPs of the symbiotic bacteria from those present on the surface of the pathogens (Korecka and Arulampalam, 2012).

The above-mentioned cells and molecules, such as immune cells differentiated under the influence of symbiotic or pathogenic microflora, cytokines, inflammatory mediators, pathogen toxins, and PAMPs after translocation into the systemic circulation, may further infiltrate the central nervous system (CNS) and influence its functioning (Zhang et al., 2021).

### 2.2. Gut microbiota and neurotransmitters interaction

The main neurotransmitters that may play a role in the gut-brain axis communication are serotonin, dopamine, noradrenaline, and gamma-aminobutyric acid (GABA) (Cox and Weiner, 2018; Makris et al., 2021). These substances are synthesized not only in the CNS but also in enteroendocrine (EEC) cells that have the ability to synthesize neurotransmitters under the influence of intestinal peptides, as well as the gut microbiota itself (Makris et al., 2021).

It is worth emphasizing that one percent of the intestinal epithelial cells are EEC cells, the role of which is to synthesize and release substances into the intestinal lumen in the presence of ingested carbohydrates, triglycerides, and proteins, and to regulate intestinal motility, secretion, and food intake (Näslund and Hellström, 2007; Gunawardene et al., 2011; Wu et al., 2013).

It has been shown that EEC cells synthesize approximately 90 percent of the total serotonin produced in the human body (Bellono et al., 2017). Moreover, an important role in the synthesis of serotonin is played by the liver expressing the enzyme tryptophan-2,3-dioxygenase (TDO, tryptophan pyrolase), degrading tryptophan to N-formylkynurenine, which is then deformylated by formidases to kynurenine. This reduces the concentration of tryptophan which may be converted to the serotonin in the brain (Wirleitner et al., 2003; Müller and Daya, 2008). In the digestive system, serotonin is involved in the activation of innate intestinal reflexes, mediating intestinal-brain communication, regulating the immune system, and has a protective/regenerative effect on neuronal cells and the interstitial cells of Cajal, but on the other hand it may cause enteritis (Mawe and Hoffman, 2013). Studies indicate that the level and activity of serotonin, synthesized both in the CNS and peripherally, is strongly influenced by the gut microbiota. In particular, a few representatives of the gut microbiota such as *Candida*, *Streptococcus*, *Escherichia*, and *Enterococcus* are capable of producing serotonin directly (Holzer and Farzi, 2014; Jameson and Hsiao, 2018). Nevertheless, it was noticed that serotonin has a significant effect on the composition and activity of the gut microbiota. Fung et al. (2019) observed that elevated intestinal serotonin levels increased the relative abundance of spore-forming bacteria.

Serotonin as well as other neurotransmitters, including dopamine and norepinephrine, are involved in gut-brain communication (Makris et al., 2021). It has been found that both dopamine and norepinephrine can be synthesized directly by the gut microbiota. Dopamine can be synthesized by the following microorganisms: *Bacillus cereus*, *Bacillus mycoides*, *Bacillus subtilis*; *Escherichia coli*; *Hafnia alvei*; *Klebsiella pneumoniae*; *Morganella morganii*; *Proteus vulgaris*; and *Staphylococcus aureus*. In turn, norepinephrine can be synthesized by: *Bacillus mycoides*, *Bacillus subtilis*; *Escherichia coli*; and *Proteus vulgaris* (Strandwitz, 2018). Moreover, gut microbiota is capable of synthesizing oxidases such as laccase, which has oxidizing properties, and catabolize catecholamines to reactive oxygen species (ROS) and dopamine quinone (DAQ), which is associated with mitochondrial dysfunction and dementia in patients with Parkinson’s disease (Sharma et al., 2018). Gut microbiota may also play a role in the transport of catecholamines (Singh et al., 2007). In addition, gut microbiota may affect the availability of tryptophan, a precursor to catecholamines (Rackers et al., 2018). These data appear to have important implications because the nucleus tractussolitarius (NTS), the main site for catecholamine synthesis, receives signals from the phrenic and vagus nerves (Paton et al., 2000). In turn, the NTS, *via* noradrenergic neurons and catecholamines, can activate the hypothalamic-pituitary-adrenal (HPA) axis, which has a significant impact on the composition and activity of the gut microbiota (Herman et al., 2016).

It has also been shown that GABA is directly synthesized by some types of intestinal bacteria, mainly: *Bifidobacterium* spp. and *Lactobacillus* spp. (Strandwitz, 2018). In addition, it was found that lactate, a substrate for the SCFAs produced by gut microbiota, through the pathway dependent on the brain G protein-coupled receptor 81 (GPR81), induces anti-GABA-transmitting effects (Caspani et al., 2019). On the other hand, the administration of *Lactobacillus rhamnosus* contributed to an increase in the expression of GABA receptors in the cingulate cortex and a decrease in the expression of GABA receptors in the hippocampus, amygdala, and the locus coeruleus, leading to a reduction in anxiety and depressive-like behavior in adult male BALB/c mice (Tanida et al., 2005; Bravo et al., 2011).

### 2.3. Gut microbiota metabolites and brain interaction

It appears that SCFAs can influence the brain indirectly by activating the immune system and the autonomic nervous system (Stilling et al., 2016). First, SCFAs are able to stimulate the activity of microglia and change the selectivity of the blood-brain barrier (BBB) permeability (Strandwitz, 2018). It is likely that the abundant exposure of H^+^ dependent monocarboxylate transporters (MCTs) in endothelial cells may facilitate the penetration of SCFAs by BBB. Clinical studies have shown the presence of SCFAs in human cerebrospinal fluid (CSF) in the following range: acetate 0–171 μM, propionate 0–6 μM, and butyrate 0–2.8 μM. In addition, the mean level of SCFAs in the human brain is 17.0 pmol/mg of tissue for butyrate, and 18.8 pmol/mg of tissue for propionate (Silva et al., 2020). The SCFAs also appear to play a significant role in maintaining the integrity of the BBB. Studies on germ-free mice showed decreased expression of tight junction proteins such as claudin and occludin in the endothelium, leading to increased BBB permeability. In turn, colonization of adult germ-free mice with complex microflora or monocolonization with SCFA-producing bacterial strains restored BBB integrity (Braniste et al., 2014).

It has also been shown that SCFAs can act indirectly on the CNS by binding SCFAs to their receptors on EEC cells, stimulating the secretion of GLP-1 and PYY, which can act on the CNS *via* the vagus nerve (Bliss and Whiteside, 2018). Another mechanism by which SCFAs respond to systemic functions is the inhibition of histone deacetylase (HDAC) activity, thereby promoting the acetylation of a lysine residue present in histones and nucleosomes in various cell populations, including the intestine, the autonomic nervous system (ANS), and the CNS (Stilling et al., 2016).

It was also found that SCFAs can affect the synthesis of neurotransmitters in the CNS, butyrate and propionate are able to stimulate the synthesis of dopamine and norepinephrine, and propionic acid can modulate serotonergic neurotransmission and affect the levels of GABA, dopamine, and serotonin (Nankova et al., 2014; El-Ansary et al., 2015; Stilling et al., 2016). The available data suggest that the SCFAs synthesized by the gut microbiota after entering the host cells (passive diffusion and/or active transport) inhibit the histone deacetylase inhibitor (HDAC) or, by interacting with membrane receptors, activate various intracellular signaling pathways that modify the expression of a given gene (Nankova et al., 2014; Stilling et al., 2016). It has been shown that butyric and propionic acids can regulate tyrosine hydroxylase (TH) mRNA levels through various transcription factors, including the activation of the cAMP-response element binding protein (CREB) and, consequently, may lead to increased production of catecholamines (Nankova et al., 2014).

Short-chain fatty acids may also modulate the expression of signaling molecules important for learning and memory, such as brain-derived neurotrophic factor (BDNF), *N*-methyl-D-aspartate receptor subtype 2B (NR2B) subunit, the serotonin transporter, and the neuropeptide Y system. It has been shown that sodium butyrate is able to stimulate BDNF expression, neurogenesis, and neuronal proliferation in rodents, and facilitate long-term consolidation (Silva et al., 2020). Moreover, SCFAs influence several nervous functions, such as the regulation of the circadian rhythm and the control of appetite (Silva et al., 2020; Williams et al., 2020; Makris et al., 2021). It appears that SCFAs may affect neuronal function through a pathway dependent on GPR41 and GPR43 receptors or HDAC inhibitory activity (Nankova et al., 2014; Patnala et al., 2017).

## 3. Gut-brain axis (GBA)

The observed concordance of the phylogenetic trees of the gut microbiota and primates indicates co-evolution of the host organism, including humans, with the resident microorganisms. Through co-evolution, the gut microbiota influenced the formation of the host’s immune system, which developed complex mechanisms for identifying and destroying microbes (Dominguez-Bello et al., 2019). Moreover, available data suggest that gut microbiota may influence host brain activities such as behavior, appetite regulation, and serotonin metabolism (Schroeder and Bäckhed, 2016). Changes in gut microbiota composition have been linked to many neurological diseases such as neurodegenerative disorders (Vogt et al., 2017). The above effects on the host brain are likely to be exerted by gut microbiota *via* the gut-brain axis (GBA). Moreover, the same pathway enables the CNS to influence the composition and activity of the gut microbiota (Ahlawat et al., 2021; Makris et al., 2021). The gut-brain axis is a bidirectional signaling pathway between the gut and the CNS (Bauer et al., 2016), and this action is possible through three GBA communication pathways: (1) immunological; (2) neuroanatomical; and (3) neuroendocrine (Wang and Wang, 2016).

### 3.1. Immunological signalization in the GBA

**FIGURE 1:**
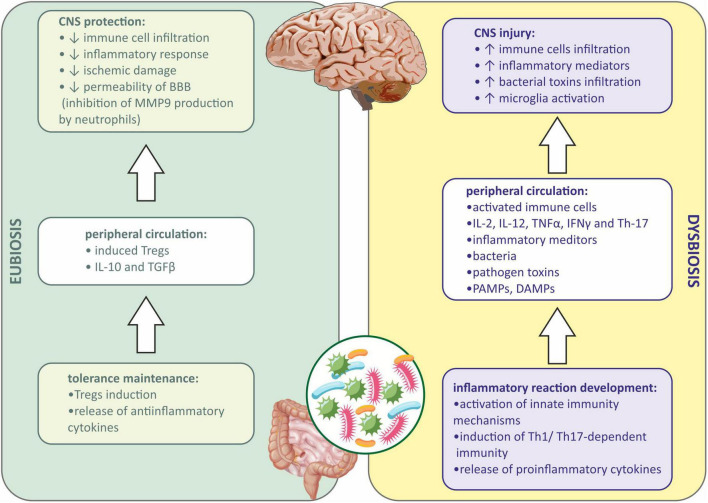
Immune signaling between gut microbiota and central nervous system during eubiosis and dysbiosis. BBB, blood-brain barrier; CNS, central nervous system; DAMPs, damage-associated molecular patterns; IFNγ, interferon gamma; IL-2, interleukin-2; IL-10, interleukin 10; IL-12, interleukin 12; MMP9, matrix metallopeptidase 9; PAMPs, pathogen associated molecular patterns; TGFβ, transforming growth factor β; Th-1, T helper 1 cells; Th-17, T helper 17 cells; TNFα, tumor necrosis factor α; Tregs, regulatory T cells.

As described above, the immune mechanisms are shifted to anti-inflammatory responses in the state of eubiosis in the gut (Lee and Kim, 2017). Tregs are generated when antigens are presented by DCs to lymphocytes in the GALT during the eubiotic state (Lathrop et al., 2011). Tregs produce anti-inflammatory cytokines, such as IL-10 and TGFβ, which are responsible for the inhibition of proinflammatory cytokine production, switching the immune response from Th1/Th17-dependent to Th2-dependent (Li et al., 2021), and therefore quenching the immune reaction and promoting the repair process in the damaged tissue (Mingomataj and Bakiri, 2016). Induced Tregs may also translocate into circulation and inhibit the inflammatory responses in the organism (Weiner and Wu, 2011). The depletion of Tregs was shown to increase CNS damage in mice after stroke (Liesz et al., 2009). It was also presented that Tregs may influence the CNS from the periphery. In rodents with ischemic stroke evoked, injection of Tregs diminished immune cell infiltration, inflammatory response, and ischemic damage of the brain. This was due to the inhibition of matrix metallopeptidase-9 (MMP-9) production by neutrophiles, which suppressed the remodeling of the BBB and decreased its permeability (Li et al., 2013; Figure 1).

When dysbiosis occurs, a local inflammatory response in the gut is observed first, and then peripheral inflammation develops when inflammatory mediators, bacteria, metabolites, PAMPs, etc., enter the systemic circulation (Lobionda et al., 2019). Both innate and adaptive immune mechanisms are involved. PAMPs *via* PRR receptors on the host cells activate innate immunity mechanisms (Bergstrom et al., 2012). In addition, the immune cells in GALT are involved during dysbiosis, and lymphocytes under the influence of inflammatory mediators and PAMPs differentiate into proinflammatory subtype (Th1 and Th17), and further produce proinflammatory cytokines, such as interleukin-2 (IL-2), interleukin-12 (IL-12), TNFα, IFNγ, and Th-17. Peripheral inflammation affects the BBB integrity, allowing the infiltration of immune cells and inflammatory mediators into the CNS (Amoo et al., 2021). Besides, bacterial toxins present in the circulation may also infiltrate into the CNS (Abdel-Haq et al., 2019; Zhang et al., 2021). Microglia, resident immune cells in the CNS may be further activated by infiltrating proinflammatory cytokines from the periphery, and sterile immune reaction may be evoked, eventually causing CNS injury (Cryan et al., 2019; Gwak and Chang, 2021; Figure 1).

**FIGURE 2:**
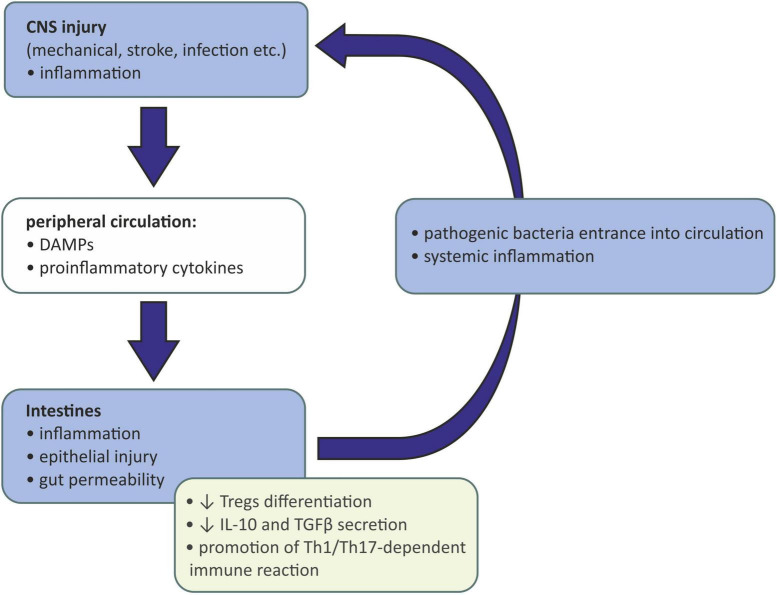
Influence of injured and inflamed central neurons system on gut microbiota. CNS, central nervous system; DAMPs, damage-associated molecular patterns; IL-10, interleukin 10; TGFβ, transforming growth factor β; Th-1, T helper 1 cells; Th-17, T helper 17 cells; Tregs, regulatory T cells.

The gut-brain immune communication acts in both ways. After injury of the CNS caused by mechanical injury, stroke, infection, etc., the damaged tissue releases damage-associated molecular patterns (DAMPs) activating resident microglia. Activated microglia of the proinflammatory (M1) phenotype release inflammatory mediators acting as chemoattractants and are responsible for recruiting peripheral immune cells to the inflammation site. Those cells, neutrophils, monocytes, and CD4^+^ T cells, also produce proinflammatory cytokines, which together with DAMPs may enter the peripheral circulation and affect the peripheral tissues (Shichita et al., 2009). If the intestinal barrier is reached, gut inflammation may be evoked, causing gut permeability, epithelial injury, and the entrance of pathogenic bacteria into circulation. This may eventually lead to systemic inflammation (Arya and Hu, 2018). Moreover, intestinal inflammation causes the reduction in Tregs differentiation and IL-10 and TGFβ secretion and the promotion of a Th1/Th17-dependent immune reaction. Such a lack of anti-inflammatory signaling and promotion of proinflammatory mechanisms further activates the immune cells and exacerbates the inflammation of the CNS (Rahman and Dandekar, 2021; Schächtle and Rosshart, 2021; Figure 2).

### 3.2. Neuroanatomicsignaling in gut microbiota-brain communication

**FIGURE 3:**
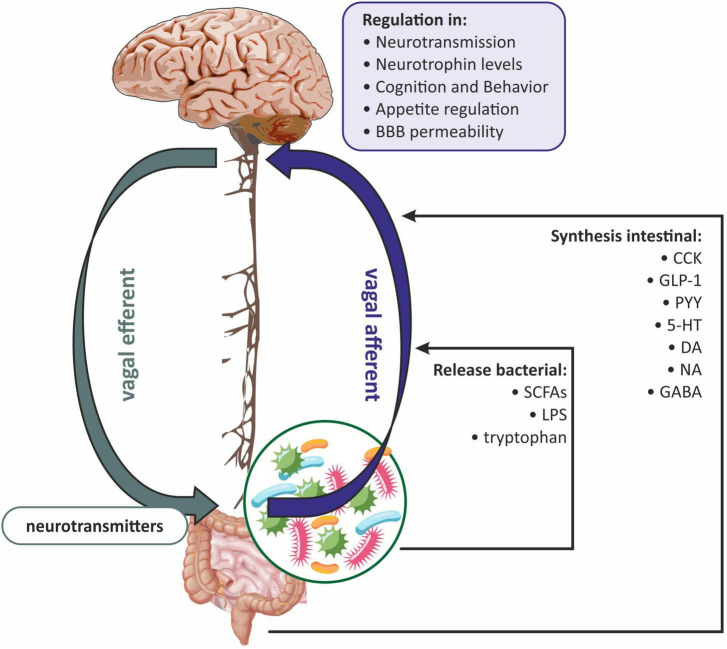
The role of the vagus nerve in gut microbiota-brain communications. BBB, blood-brain barrier; CCK, cholecystokinin; DA, dopamine; GABA, gamma-aminobutyric acid; GLP-1, glucagon-like peptide-1; 5-HT, serotonin; LPS, lipopolysaccharide; NA, noradrenaline; PYY, peptide YY (PYY); SCFAs, short-chain fatty acids.

Based on the available data, it can be concluded that the gut microbiota-induced vagal signaling affects the critical immune components of the microbiota-gut-brain axis and allows the vagus nerve to be seen as an integral part of the bidirectional neuroimmunoendocrine pathway (Liu et al., 2021; Figure 3). Therefore, two pathways can be distinguished at the neuroanatomical level. The first direct way between the GI and the brain consists of the vagus nerve and the ANS. While the second indirect way is made by the connection between the ANS and the enteric nervous system (ENS) (Wang and Wang, 2016).

It has been proven that the vagus nerve plays a key role in the communication between the gut microbiota and the brain *via* anatomical signaling (Czerwińska et al., 2021; Makris et al., 2021). Some researchers believe that the vagus nerve innervates the entire digestive tract, while others argue that it only innervates to the left colon flexion (Bonaz et al., 2018). Vagal afferent fibers are distributed in all layers of the gastrointestinal wall but do not penetrate the epithelial layer into the lumen of the intestine (Wang and Powley, 2007). The afferent endings of the vagus nerve have been divided into three subtypes: (1) afferent endings located at the apices of the intestinal villi directly under the epithelial wall; (2) afferent endings around the intestinal glands or crypts located below the crypt-villus junction; (3) afferent endings along the antral glands of the stomach (afferent endings of the antral gland) forms the end concentrations directly below the luminal epithelial wall (Powley et al., 2011). All of the above vagus nerve endings are both chemosensitive and mechanosensitive (Bonaz et al., 2018). Vagus nerve chemoreceptors are presumed to be involved in communication between the gut microbiota and the brain by detecting SCFAs and/or intestinal peptides (Raybould, 2010; Figure 3). Oleate (one of the SCFAs) acts on the vagal afferent fibers *via* cholecystokinin (CCK), while butyrate can directly activate the vagus nerve (Lal et al., 2001). Similarly, the LPS synthesized by the gut microbiota can interact with the TLRs located on the vagus nerve fibers at the nodose ganglion level (Hosoi et al., 2005; Figure 3).

The vagus nerve can also receive signals from the gastrointestinal tract indirectly, involving the ENS, which is part of the autonomic nervous system. The ENS consists mainly of enteric glial cells (EGCs), which resemble astrocytes in the CNS (Ahlawat et al., 2021). The ENS is distributed throughout the intestinal wall, including the lamina propria of the mucosa (Obata and Pachnis, 2016; Kho and Lal, 2018). From the ganglia of the ENS, the neuron fibers go to the prevertebral ganglia and then to the spinal cord at the level of the T5-L2 and S2-S4 segments, and to the vagal nuclei. Afferent fibers of the vagus nerve and spinal cord concentrate hormonal and mechanical stimuli in the NTS and in the dorsal motor nucleus, where the signal is integrated, and next sent to the hypothalamus, as well as to the basal ganglia and brain stem nuclei (Sobrino Crespo et al., 2014; Bauer et al., 2016; Zhao et al., 2018). NTS signaling is mediated by proopiomelanocortin (POMC), catecholaminergic neurons, and N-methyl-D-aspartate (NMDA) glutamate receptors (Bauer et al., 2016; Bliss and Whiteside, 2018). The vagus nerve can also receive information from the intestinal lumen *via* enteroendocrine (EEC) cells, which make up one percent of all intestinal epithelial cells (Bonaz et al., 2018). It has been shown that EEC can release serotonin (5-HT), which then activates 5-HT3 receptors on the vagal afferent fibers (Li et al., 2000). EEC effects on the vagus nerve may also be indirect through intestinal peptides such as CCK, glucagon-like peptide-1 (GLP-1), and peptide YY (PYY) (Strader and Woods, 2005). In general, gut hormones fall into two broad categories: orexigenic, for example, ghrelin, which together with neuropeptide Y (NPY) and agouti-related peptide (AGRP) neurons, increases hunger; and anorexigenic, i.e., appetite-suppressing peptides such as GLP-1, PYY, and CCK (Weltens et al., 2018). The vagus nerve has receptors for both anorexigenic and orexigenic intestinal peptides (Strader and Woods, 2005). Subsequently, the signal is sent to the CNS, ultimately leading to the modulation of reward regions (amygdala and nucleus accumbens) and appetite regulation (Weltens et al., 2018; Czerwińska et al., 2021; Figure 3).

Evidence for the direct influence of the gut microbiota on the activation of the vagus nerve is provided by a few experimental studies. *Ex vivo* studies have shown that the application of *Lactobacillus johnsonii* to the isolated fragment of the jejunum contributed to an increase in the firing rate of the vagus nerve fibers, which was prevented by the previous subdiaphragmatic vagotomy (Perez-Burgos et al., 2013). It was reported that duodenal injection of *Lactobacillus johnsonii* in male Wistar rats caused an increase in gastric activity of the vagus nerve (Tanida et al., 2005). It was also shown that signals sent by the gut microbiota *via* the vagus nerve can go directly to the CNS. Male mice receiving *Lactobacillus rhamnosus* for 14 days showed a reduction in anxiety-like behavior and a decrease in the HPA axis activity. Whereas vagotomy in these mice abolished the anxiolytic effects induced by *Lactobacillus rhamnosus* (Liu et al., 2021). Bravo et al. (2011) observed that chronic oral administration of *Lactobacillus rhamnosus* (JB-1) in healthy adult male BALB/c mice increased gamma-aminobutyric acid (GABA) expression in the cingulate cortex and decreased GABA in the hippocampus, amygdala, and locus coeruleus. Interestingly, the above effects were abolished by vagotomy. In addition, studies in healthy Sprague Dawley rats have shown that one of the bacterial metabolites, indole, can stimulate the ECC of the colon to secrete GLP-1, which in turn stimulated colonic vagal afferent activity (Buckley et al., 2020). Recently, Tashtush et al. (2022) showed that administration of fecal supernatant from patients with active inflammatory bowel disease (IBD) on the C57/Bl6 mouse vagal afferent neurons (nodose ganglion; NG) increased their excitability, possibly due to mediators such as cysteine protease, activating protease-activated receptor 2 (PAR2) dependent signaling pathways, which leads to the inhibition of voltage-gated K^+^ currents.

Moreover, available data have shown that gut microbiota can affect the nervous system of the host by altering the metabolism of neurotransmitters. Studies (Engevik et al., 2021) on adult germ-free Swiss Webster mice treated with live *Bifidobacterium dentium via* oral gavage showed that bacterial-produced acetate contributed not only to increased 5-HT release from the EEC, but also increased 5-HT2a receptor expression in the hippocampus and lowered the anxiety-like behaviors in the tested mice. In turn, a study (Peng et al., 2022) revealed that another bacterial metabolite, succinate, has a protective effect on dopaminergic neurons in the substantia nigra. In addition, it was shown that oral supplementation of the three major SCFAs (acetate, propionate, and butyrate) in C57BL/6J mice undergoing psychosocial stress reduced disturbance in food-seeking behavior as well as reducing anti-depressant and anti-anxiety effects (van de Wouw et al., 2018). SCFAs are presumed to affect the host nervous system in a hormone-like fashion *via* specific G-protein coupled receptors (GPCRs) (Barki et al., 2022), which also include many metabotropic neurotransmitter receptors. On the other hand, emotional disorders such as chronic stress not only affect the metabolism of neurotransmitters but also have strong implications on the composition of the gut microbiota (Yang et al., 2021). Moreover, norepinephrine, a stress-related hormone has been shown to increase the abundance of *Desulfovibrio vulgaris*. A significant population increase of this bacterium is observed in patients with inflammatory bowel disease and irritable bowel syndrome (Coffman et al., 2022).

### 3.3. Neuroendocrine signaling in the gut microbiota-brain communication

**FIGURE 4:**
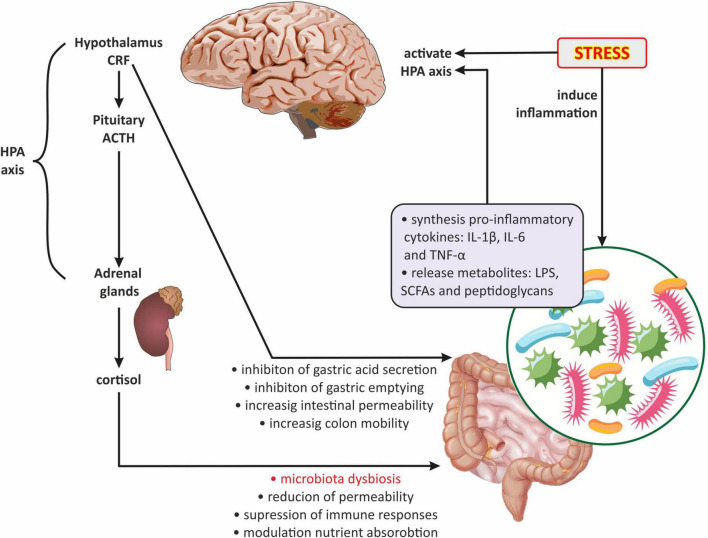
The role of the hypothalamic-pituitary-adrenal axis in gut microbiota-brain communications. ACTH, adrenocorticotropic hormone; CRF, corticotropin-releasing factor; HPA axis, hypothalamic-pituitary-adrenal axis; IL-1β, interleukin-1 beta; IL-6, interleukin 6; LPS, lipopolysaccharide; SCFAs, short-chain fatty acids; TNFα, tumor necrosis factor α.

The neuroendocrine level primarily includes the HPA axis, which plays a key role in the stress response and is also one of the main components of the gut-brain axis. The HPA axis begins in the paraventricular nucleus of the hypothalamus, where the corticotropin releasing factor (CRF) is synthesized. In turn, CRF stimulates the pituitary gland to produce the adrenocorticotropic hormone (ACTH), which leads to the release of glucocorticoids (cortisol and corticosterone) from the adrenal cortex (Misiak et al., 2020). In addition, CRF stimulates the locus coeruleus to synthesize catecholamines, thus leading to an increase in the noradrenergic activity of the brain (Jedema and Grace, 2004). There is evidence that the gut microbiota develops in parallel with the HPA axis, moreover, they are in constant communication (de Weerth, 2017; Frankiensztajn et al., 2020; Misiak et al., 2020; Williams et al., 2020). Interestingly, the two-way communication between the gut microbiota and the HPA axis is increasingly emphasized (Dinan and Cryan, 2017; Foster et al., 2017; Morris and Ridlon, 2017; Figure 4). It has been reported that the abnormal formation of the HPA axis during brain development may affect microbial colonization and visceral sensitivity (Pellissier and Bonaz, 2017). Irritable bowel syndrome studies have shown that cortisol can directly activate resident immune cells and external primary afferent fibers in the gastrointestinal tract (Moloney et al., 2016). Moreover, both experimental and clinical researchers have demonstrated that the stress-related HPA axis response can increase intestinal permeability leading to dysbiosis (Vicario et al., 2012; Vanuytsel et al., 2014; Figure 4).

Significant information on the existence of interactions between the gut microbiota and the HPA axis was provided by Sudo et al. (2004). These researchers demonstrated that germ-free mice (GF; mice raised in the absence of microbes) were more sensitive to restraint stress than mice with normally functioning microbiota but in the absence of specific pathogens (SPF; specific pathogen-free mice). GF mice also showed decreased levels of cortical glucocorticoid receptor mRNA expression and increased levels of CRF mRNA and protein in the hypothalamus compared with SPF mice. Moreover, the same researchers observed that the increase in plasma ACTH and corticosterone levels in response to restrictive stress was significantly greater in GF mice compared with SPF mice. Probably the observed differences in stress response between GF and SPF mice could also be caused by decreased expression of POMC and encoding CRF receptor type 1 (Crhr1) genes (Vagnerová et al., 2019).

Experimental studies have also shown that numerous stressors causing an increase in the activity of the HPA axis may affect the development of dysbiosis (Dubinski et al., 2021). It has been shown that chronic s.c. injection of ACTH hormone fragment in female Wistar rats not only caused their depressive-like behavior, but also caused changes in the community of gut microbiota, namely a marked increase in *Ruminococcus* and *Klebsiella* and a reduction in the population of *Akkermansia* and *Lactobacillus* (Song et al., 2019). Wu et al. (2020) in research carried out on rats with stress-induced hypertension (SIH) noted a reduction in HPA axis hyperactivity and blood pressure due to the administration of an antibiotic cocktail containing ampicillin, vancomycin, neomycin, and metronidazole. Catecholamines released in response to stress probably play an important role in this process. It was proven that catecholamines can stimulate the growth of gram-negative bacteria (Lyte and Ernst, 1992). The above data seem to confirm studies conducted in patients suffering from irritable bowel syndrome (IBS) in combination with emotional distress including anxiety and depressive symptoms. In these patients, already slightly lower concentrations of serotonin and norepinephrine corresponded to significant changes in the composition of gut microbiota. Namely, serotonin levels were positively correlated with the abundance of *Proteobacteria*, and norepinephrine was positively correlated with *Bacteroidetes* and negatively correlated with *Firmicutes* (Barandouzi et al., 2022). Similarly, clinical data confirmed that stress and the accompanying increased cortisol blood level have a significant impact on the development of gut dysbiosis (Misiak et al., 2020).

In contrast, both experimental and clinical studies revealed that probiotics based on *Bacillus licheniformis*, *Saccharomyces boulardii*, *Lactobacillus rhamnosus*, and *Bifidobacterium* spp. contributed to the inhibition of stress-induced HPA axis hyperactivity, as well as alleviated depressive-like behavior and anxiety-related behavior (Eutamene et al., 2007; Gareau et al., 2007; Desbonnet et al., 2010).

It appears that the gut microbiota can act on the HPA axis through several mechanisms (Figure 4): (1) gut dysbiosis contributes to the increased release of pro-inflammatory cytokines, including IL-1, IL-6, and TNF-α which can cross the BBB and activate the HPA axis (Turnbull and Rivier, 1995; Banks, 2005); (2) the HPA axis can be activated by bacterial metabolites such as LPS, SCFAs, and peptidoglycans (components of bacterial cell walls) (Arentsen et al., 2017; van de Wouw et al., 2018); (3) by the influence of gut microbiota on the HPA axis *via* the vagus nerve, affecting the NTS activity of noradrenergic neurons (Paton et al., 2000; Herman et al., 2016); and (4) by changes in HPA axis activity caused by the modulation of central gene expression in the hippocampus and hypothalamus by gut microbiota (Frankiensztajn et al., 2020).

## 4. The role of the gut-brain axis in the treatment of CNS diseases

Currently, many researchers emphasize the possibility of using the gut-brain axis in the treatment of many neurological diseases such as autism spectrum disorder (ASD), Alzheimer’s disease (AD), and Parkinson’s disease (PD) (Metta et al., 2022; Taniya et al., 2022; Varesi et al., 2022).

Clinical studies increasingly point to a link between ASD and intestinal dysfunction. It is estimated that up to 70% of children with ASD have impaired function of the digestive tract (Sajdel-Sulkowska et al., 2019). In the case of gut microbiota, these abnormalities concern the development of an excessive number of pathogenic bacteria such as *Clostridium tetani* (Shaw, 2010). In turn, treating children with ASD with anti-clostridium antibiotics resulted in a decrease in typical behaviors for them (Kang et al., 2017). Moreover, high hopes are attached to Microbiota Transfer Therapy (MTT) in the treatment of patients with ASD (Taniya et al., 2022).

Currently, the role of disorders in the functioning of the gut brain-axis in the pathogenesis of neurodegenerative diseases such as AD and PD is increasingly emphasized. Damage to the intestinal barrier is hypothesized to lead to a systemic inflammatory response, which in turn impairs BBB function and promotes neuroinflammation leading to neurodegeneration and neuron damage. Consequently, damaged GBA potentiates β-amyloid deposition in AD and misfolding and aggregation of α-synuclein in PD (Quigley, 2017). Similar to patients with ASD, also in the case of therapy of patients with neurodegenerative diseases, much attention is paid to the need to restore the proper composition of the gut microbiota with fecal microbiota transplantation (FMT) or probiotics (Metta et al., 2022; Varesi et al., 2022). It is likely that FMT has a beneficial effect on reducing symptoms in patients with PD through neuroprotective effects against toxicity induced by the TLR4/TNF-α signaling pathway and 1-methyl-4-phenyl-1,2,3,6-tetrahydropyridine (MPTP) (Metta et al., 2022). So far, few clinical trials have shown the beneficial effect of FMT on improving cognition, memory, and mood, as well as gut microbiota biodiversity and SCFA production in patients with AD (Hazan, 2020; Park et al., 2021). However, the results of studies on the effect of probiotics on symptom improvement in patients with AD are inconclusive. Akbari et al. (2016) showed that after 12 weeks of daily administration of a mixture of *Lactobacillus acidophilus*, *Lactobacillus casei*, *Bifidobacterium bifidum*, and *Lactobacillus fermentum*, AD patients showed a significant improvement in mini-mental state exam results. On the other hand, the administration of two different probiotic mixtures: one containing *Lactobacillus fermentum*, *Lactobacillus plantarum*, and *Bifidobacterium lactis* and the other containing *Lactobacillus acidophilus*, *Bifidobacterium bifidum*, and *Bifidobacterium longum*, did not contribute to the improvement of cognitive functions in patients with severe Alzheimer’s disease (Agahi et al., 2018). Moreover, numerous studies indicate a well-chosen diet as a quick way to modify the composition and metabolism of the gut microbiota, reduce inflammation, and help maintain eubiosis and proper dependencies in the gut-brain axis (Metta et al., 2022; Varesi et al., 2022).

## 5. Conclusion

Gut microbiota, which is an integral part of the human body, is able to summon and control many of its physiological processes. The host organism also has great influence on the composition and activity of the gut microbiota. Recent studies show that the gut-brain axis is a well-established concept, indicating the bidirectional cooperation between two organs of the human body, the brain, and the gut, and in particular the microbiota residing in the intestines. Many studies have shown the engagement of different routes of communication able to transmit information between the two separate organs, such as immune, nervous, and endocrine. It was also proved that disturbances in one of the organs may affect the proper functioning of the other, also in a bidirectional manner. Unfortunately, the routes for signal transduction involved in the gut-brain communication are still not fully known as well as what roles are played by inflammation, neurotransmitters, intestinal peptides, and bacterial metabolites. In addition, a significant amount of information on the gut-brain axis activity comes from studies on animal models, which, while providing relevant information, should not be directly extrapolated to the human population. Therefore, more research is needed to elucidate the importance of gut microbiota not only for adult organisms, but also for developing ones, with the target of preventing or treating CNS diseases.

## Author contributions

KK and KC: methodology, resources, and writing—original draft preparation. KC and AC-J: supervision. KC: project administration. All authors conceptualization, writing—review and editing, read and agreed to the published version of the manuscript.
